# The deubiquitinating enzymes USP4 and USP17 target hyaluronan synthase 2 and differentially affect its function

**DOI:** 10.1038/oncsis.2017.45

**Published:** 2017-06-12

**Authors:** M Mehić, V K de Sa, S Hebestreit, C-H Heldin, P Heldin

**Affiliations:** 1 Ludwig Institute for Cancer Research, Science for Life Laboratory, Uppsala University, Uppsala, Sweden; 2 Department of Genomics and Molecular Biology, International Research Center, A.C. Camargo Cancer Center, São Paulo, Brazil; 3 Department of Pathology, Faculty of Medicine, University of São Paulo, São Paulo, Brazil; 4 Department of Medical Biochemistry and Microbiology, Uppsala University, Uppsala, Sweden

## Abstract

The levels of hyaluronan, a ubiquitous glycosaminoglycan prominent in the extracellular matrix, is balanced through the actions of hyaluronan-synthesizing enzymes (HAS1, 2 and 3) and degrading hyaluronidases (Hyal 1, 2, 3 and PH20). Hyaluronan accumulates in rapidly remodeling tissues, such as breast cancer, due to deregulated expression of the *HAS2* gene and/or alterations of HAS2 activity. The activity of HAS2 is regulated by post-translational modifications, including ubiquitination. In order to identify deubiquitinating enzymes (DUBs) that are involved in de-ubiquitination of HAS2, a complementary (cDNA) library of 69 Flag-HA-tagged human DUBs cloned into retroviral vectors was screened in human embryonic kidney (HEK) 293T cells for their ability to de-ubiquitinate myc-tagged HAS2. Several DUBs were found to decrease the ubiquitination of 6myc-HAS2, among which, the most effective were USP17 and USP4. USP17 efficiently removed polyubiquitination, whereas USP4 preferentially removed monoubiquitination of 6myc-HAS2. Co-immunoprecipitation studies revealed interactions between HAS2 and USP17, as well as between HAS2 and USP4, in membrane preparations of HEK293T cells. USP17 significantly stabilized 6myc-HAS2 protein levels, whereas USP4 did not. The silencing of USP17 led to decreased hyaluronan production, whereas the suppression of USP4 increased hyaluronan synthesis. Importantly, high levels of USP17 and HAS2 were detected in a panel of cancer cell lines compared to normal cells, and immunohistochemical stainings revealed higher expression of USP17 and HAS2 in tissues of lung cancer patients compared to normal tissue. In conclusion, USP17 and USP4 differently affect HAS2 ubiquitination, and the stability and function of HAS2.

## Introduction

Hyaluronan is a non-sulfated glycosaminoglycan that is synthesized by three related hyaluronan synthases (HAS1, HAS2 and HAS3) that exhibit 55–71% sequence similarity.^1, 2, 3, 4, 5^ The newly synthesized hyaluronan is directly extruded through the plasma membrane and stabilize pericellular and extracellular matrices. Hyaluronan binds to receptors at the cell surface, for example, CD44, which initiates signaling events.^6, 7, 8, 9^

In addition to its presence in the extracellular space, hyaluronan has been detected, by immunohistochemical staining, in the rough endoplasmatic reticulum, the cytoplasm and the nuclei of cultured cells as well as cells in tissues.^10, 11, 12, 13^ Notably, several intracellular hyaluronan-binding proteins have been reported including the intracellular form of the receptor for hyaluronan-mediated motility,^10, 14, 15^ the vertebrate homolog of the cell cycle control protein CDC37,^16^ and the cytokine-inducible deubiquitinating enzyme DUB3/USP17, the proper expression of which is crucial for balanced cell cycle progression.^17, 18, 19, 20^

During rapid tissue remodeling during embryonic development, fibrosis, wound healing or malignant transformation, the synthesis of hyaluronan increases markedly. In such conditions, a plethora of growth and inflammatory effectors promote the transcriptional induction of the three *HAS* genes, in particular the *HAS2* gene.^21, 22^ It has been demonstrated that the *Has2* knockout mice die because of severe cardiac defects.^23^ Moreover, amplification of the *HAS2* gene predisposes to periodic feber syndrome in chinese shar-pei dogs,^24^ and HAS2 is required for TGFβ-induced epithelial to mesenchymal transition (EMT).^25^ Furthermore, HAS2-synthesized hyaluronan promotes the progression and metastasis of breast cancer cells to bone.^26, 27, 28^ Paradoxically, high molecular weight hyaluronan confers cancer resistance to naked mole rats.^29^ The diverse roles of hyaluronan are fine-tuned by hyaluronan-degrading enzymes and free radicals that can trigger the fragmentation of hyaluronan.^30, 31, 32^ Moreover, the activity of HAS2 is affected by post-translational modifications, including phosphorylation, O-GlcNAcylation and ubiquitination.^33, 34, 35, 36, 37, 38, 39, 40^ Recently, we have demonstrated that the activity of HAS2 is promoted by dimerization and is lost when the monoubiquitination site Lys190 is mutated.^36^ Notably, Lys190 is located in the glycosyltransferase domain of HAS2 and is conserved among all the three HAS isoforms.^41^

Different types of ubiquitination controls different functions, such as protein degradation, cell proliferation, apoptosis, cell cycle progression and DNA repair. Hence, deregulation of ubiquitin pathways can result in the development of human disease, including cancer.^42, 43, 44^ Polyubiquitination via Lys48 in ubiquitin promotes proteasomal degradation.^45, 46^ Monoubiquitination of proteins at one or several lysine residues regulates their intracellular location and activity.^47^ A single ubiquitin moiety is sufficient to trigger internalization of membrane proteins and delivery of cargo to multivesicular bodies leading to their fusion with lysosomes.^44, 48, 49^ Other functions of monoubiquitination include regulation of histones, DNA repair pathways, proteasomal degradation and signaling pathways, leading to apoptosis, migration and proliferation.^42, 50, 51^

The ubiquitination of proteins is reversed by DUBs. The human genome encodes ~90 DUBs, which are divided into five families: ubiquitin C-terminal hydrolases, ubiquitin-specific proteases (USPs), ovarian-tumor proteases (OTUs) and Machado-Joseph Disease protein domain proteases, all being cysteine proteases, whereas the JAMM motif protease family encodes metalloproteases.^51, 52, 53, 54^

A large number of studies have implicated HAS2 expression in inflammation^21, 24^ and tumor progression,^20, 25, 27, 55, 56, 57, 58^ and excessive accumulation of hyaluronan at inflammatory sites and in cancer has been connected to poor prognosis. The underlying mechanisms for hyaluronan accumulation are, however, not well understood. In order to further our knowledge on the regulation of HAS2 activity and stability, we have employed a retroviral expression library of 69 Flag-HA-tagged DUBs^59^ in order to identify DUBs able to de-ubiquitinate HAS2. The DUBs USP17 and USP4 were identified to efficiently remove the ubiquitination of HAS2 as well as to affect HAS2 stability and function.

## Results

### A DUB cDNA expression screen identifies USP17 and USP4 as de-ubiquitinases for HAS2

**Figure 1:**
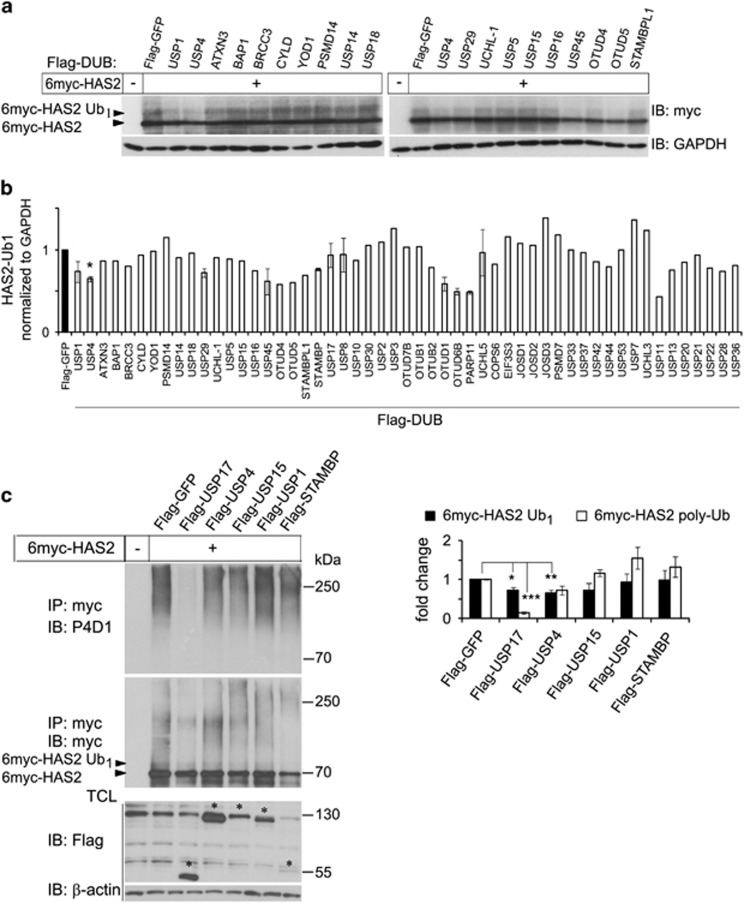
Identification of USP4 and USP17 as de-ubiquitinases of HAS2 by a DUB cDNA expression screen. (**a**) HEK293T cells (0.3 × 10^6^ cells per well in six-well plates) were co-transfected with 6myc-tagged HAS2 cDNA and individual Flag- and HA-tagged DUB cDNAs. 6myc-tagged empty vector and Flag-tagged vector encoding GFP were used as controls and to equalize the DNA load. Cell lysates were subjected to SDS–PAGE followed by immunoblotting with antibodies against myc as described in Materials and methods, to detect immunoreactive myc-tagged monoubiquitinated HAS2 (HAS2-Ub_1_; seen as a band of 5–10 kDa higher molecular mass than the 6myc-HAS2 band). (**b**) Quantification of HAS2-Ub_1_ of the immunoblots, using ImageJ. Asterisk indicates *P*<0.05 calculated with Student’s *t*-test (*n*=5), and error bars are the average of two experiments. (**c**) Re-screening of a subset of the DUBs described in **a** to determine the effects on polyubiquitination of HAS2; denaturated cell lysates were after dilution subjected to immunoprecipitation using a myc antibody followed by immunoblotting using the P4D1 antibody to detect polyubiquitinated HAS2. The right panel shows quantification of mono- as well as polyubiquitinated HAS2 after coexpression of different DUBs. Average±s.e.m. of three independent experiments is depicted. *P<0.05, **P<0.01 and ***P<0.0001, calculated with Student’s *t*-test.

In order to identify DUB enzyme(s) capable of deubiquitinating 6myc-HAS2, we expressed 6myc-HAS2 and individual DUBs from a library of 69 Flag-HA-tagged DUBs in HEK293 cells. Cell lysates were subjected to immunoblotting using a myc antibody. The monoubiquitination of HAS2 at Lys190, visualized as a 5–10 kDa mobility shift ion sodium dodecyl sulfate–polyacrylamide gel electrophoresis (SDS–PAGE),^36^ was decreased in cultures overexpressing certain DUBs (Figure 1a). Quantification of the obtained immunoblots revealed that USP4 selectively decreased monoubiquitination of 6myc-HAS2 (Figure 1b). Other DUBs, including USP1, USP29, USP45, OTUD1, OTUD6B, PARP11 and USP11, also decreased the HAS2 band representing monoubiquitination to some extent, but were not studied further (Figures 1b and c). The ectopic expression of DUBs was verified by immunoblotting using a Flag antibody (not shown) and GAPDH was used as a loading control.

To further explore candidate DUBs involved in de-ubiquitination of HAS2, we re-screened a subset of the DUBs using an *in vivo* ubiquitination assay in human embryonic kidney (HEK) 293T cells (Figure 1c and Supplementary Figure 1S). To dissociate all non-covalent interactions between HAS2 and possible interacting ubiquitinated proteins, cell lysates were prepared using a denaturation buffer. The lysates were then diluted and subjected to immunoprecipitation with anti-myc antibodies, followed by SDS–PAGE and immunoblotting with the anti-ubiquitin antibody P4D1, which recognizes both monoubiquitin and polyubiquitin chains. In control cells, transfected with only HAS2, a smeary high molecular mass band, representing polyubiquitinated HAS2, was observed. USP17 significantly removed polyubiquitination (*P*<0.0001) and less efficiently monoubiquitination, from HAS2 (*P*<0.05; Figure 1c and Supplementary Figure 1S). Notably, USP4 removed monoubiquitination (*P*<0.01) more efficiently than polyubiquitination (Figures 1b and c). USP1 removed the monoubiquitination from HAS2 during the initial DUB screening (Figures 1a and b); however, it did not significantly affect the ubiquitination status of HAS2 during the *in vivo* ubiquitination assay (Figure 1c). In view of this initial characterization, as well as the knowledge that USP4 inhibition suppresses TGFβ-induced EMT,^60^ which requires the expression of HAS2 (ref. 25) and that USP17 interacts with intracellular hyaluronan, USP4 and USP17 were chosen for further studies.

### HAS2 interacts with USP17 and USP4

**Figure 2:**
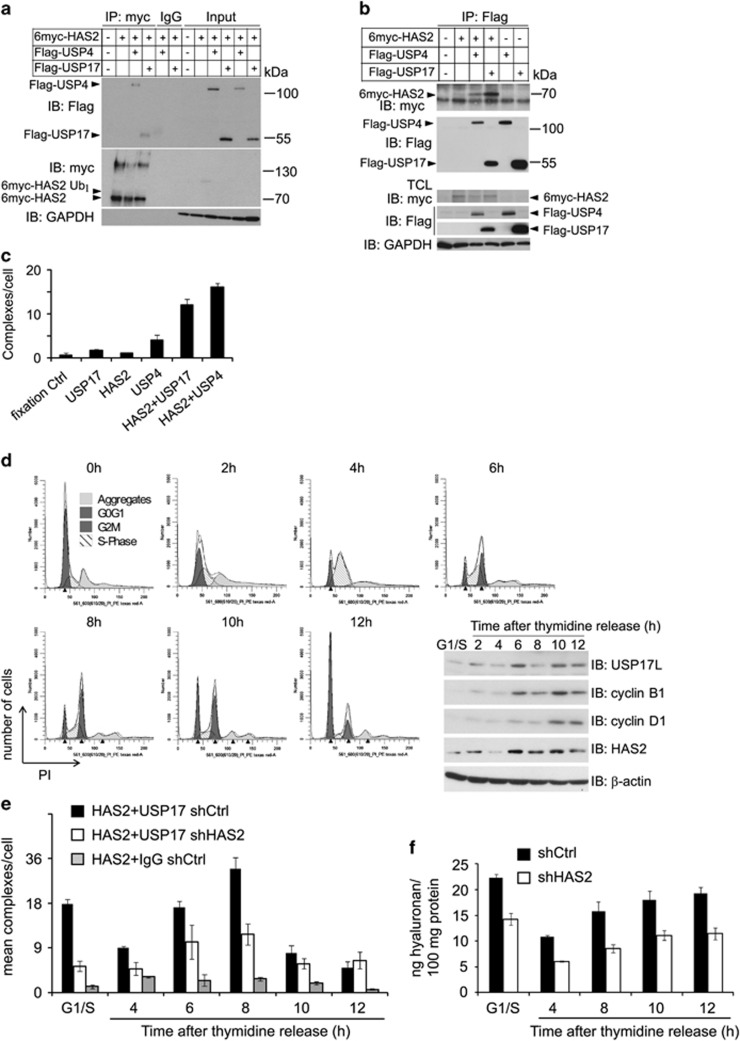
HAS2 interacts with USP4 and USP17, and the interaction between USP17 and HAS2 is cell cycle-dependent. Aliquots of membrane fractions from HEK293T cells overexpressing Flag-tagged USP4 or USP17 with 6myc-tagged HAS2 were subjected to immunoprecipitation with a c-myc (**a**) or Flag (**b**) antibodies or IgG control, and proteins were separated by SDS–PAGE. Immunoblotting was performed with Flag-M2 or c-myc antibodies; whole-membrane lysates were run in parallel. (**c**) PLA was performed in MDA-MB-231-BM cells to detect the number of endogenous HAS2–USP17 and HAS2-USP4 complexes (presented by fluorescent dots) per cell. Number of dots detected when one of the primary antibodies were omitted represent background signals. (**d**) MDA-MB-231-BM cells were synchronized by double thymidine block, as described in Materials and methods; after release cells were analyzed for their cell cycle profile by FACS analysis, and the expression of HAS2, USP17, cyclin D1 and cyclin B1 were determined by immunoblotting. PLA (**e**) and hyaluronan (**f**) assays were performed at different time periods after release of MDA-MB-231-BM cells from the thymidine block. The number of fluorescent dots, representing complexes between HAS2 and USP17, and the levels of hyaluronan released, were quantified. (**a**–**d**, **f**) Representative experiments out of two independently performed, whereas **e** is the mean of two experiments±variation.

To investigate the possibility that USP4 and USP17 form complexes with HAS2, we performed co-immunoprecipitation experiments. Samples from membrane fractions prepared from HEK293T cells expressing 6myc-HAS2 and Flag-USP4 or Flag-USP17 were immunoprecipitated with the anti-myc or anti-Flag antibodies, followed by SDS–PAGE and immunoblotting with anti-Flag (Figure 2a) or anti-myc (Figure 2b) antibodies, respectively. The analysis revealed bands of 113 and 55 kDa, i.e., the expected sizes of USP4 and USP17 (Figure 2a), and of ~70 kDa (Figure 2b), which is the expected size of 6myc-HAS2, suggesting the formation of complexes between HAS2 and USP4, as well as between HAS2 and USP17. No bands were detected when 6myc-HAS2 was transfected together with Flag-GFP as a control (Figures 2a and b), when immunoprecipitation was performed with control mouse IgG (Figure 2a), or when Flag-USP4 or Flag-USP17 were transfected with empty vector (Figure 2b). Immunoblotting of whole-cell lysates with a myc antibody revealed bands of 130–140 and 78 kDa in addition to the 70 kDa 6myc-HAS2 band (Figure 2a), confirming the presence of HAS2 dimers and monoubiquitinated forms (Figure 2a).^36^ Notably, the oligomeric structures of HAS2 were absent in USP17 and USP4 immunoprecipitated fractions (Figure 2b), suggesting that the DUBs do not form complexes with oligomeric HAS2.

To confirm complex formation of endogenous components, proximity ligation assay (PLA) was performed using the breast cancer cell line MDA-231-BM, known to produce high levels of hyaluronan.^27^ Using antibodies against HAS2 and USP4 or USP17, reactive signals in the form of red dots indicating interactions between endogenous HAS2 and USP17, and HAS2 and USP4, were observed; only a low background level of dots was seen when each antibody was used alone, or in the fixation control without antibody (Figure 2c). Thus, both co-immunoprecipitation and PLA analyses supported the notion that HAS2 forms complexes with USP17 and USP4.

### The interaction between USP17 and HAS2 is cell cycle-dependent

Given that intracellular hyaluronan is associated with mitosis^10, 61^ and the expressions of USP17 and HAS2 are required for cell cycle progression,^19, 62^ we analyzed the interaction between USP17 and HAS2 in different phases of the cell cycle. The hyaluronan-producing cell line MDA-MB-231-BM was synchronized to the G1/S phase by double thymidine block. Following release of the block, cells entered the S phase within 4 h, underwent mitosis between 6 and 10 h and re-entered the G1 phase by 12 h, as demonstrated by fluorescence-activated cell sorting (FACS) analysis of propidium iodide-stained cells (Figure 2d). Analysis of the cell cycle regulatory proteins cyclin D1 and B1 by immunoblotting verified the traverse through the cell cycle, as determined by FACS analysis. Interestingly, examination of USP17 and HAS2 expression at each cell cycle phase demonstrated a concomitant and abundant expression 6–10 h after thymidine release. Endogenous HAS2–USP17 complexes detected by the PLA analysis were abundantly formed in synchronized G1/S-released cells, decreased as the cells entered the S phase and were abundantly formed again as the cells entered the G2/M and mitosis phases to markedly decrease upon cell cycle completion (Figure 2e). As expected, HAS2 knockdown to ~50% by short hairpin RNA (shRNA; #3) resulted in a decrease in the complexes (Figure 2e); similar results were also obtained by using another shRNA targeting HAS2 construct (#4; data not shown). Importantly, the hyaluronan levels released to the media were consistent with the expression levels of HAS2 and USP17, as well as their complex formation during the cell cycle phases (Figure 2f).

### The catalytic activities of USP17 and USP4 are important for HAS2 de-ubiquitination, but not for their interactions with HAS2 and the K190R mutant HAS2

**Figure 3:**
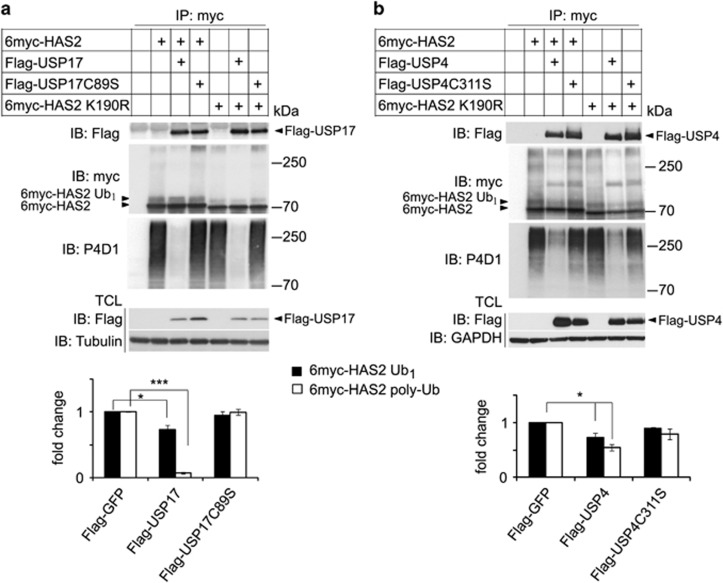
Catalytically inactive USP17 and USP4 interact with, but do not de-ubiquitinate HAS2. HEK293T cells were co-transfected with 6myc-HAS2 or its K190R mutant, and wild-type USP17 or the catalytically inactive C89S mutant USP17 (**a**), as well as wild-type USP4 or the catalytically inactive C311S mutant Flag-USP4 (**b**). 6myc-tagged empty vector and Flag-tagged vector encoding GFP were used as control and to equalize the DNA load. HAS2 was immunoprecipitated, after denaturation, with a c-myc antibody, followed by SDS–PAGE and immunoblotting with the P4D1 antibody to detect mono- and polyubiquitination, and with the myc antibody to determine total HAS2 levels. Whole-cell lysates were probed with Flag-M2 antibody to verify DUB expression, and GAPDH or tubulin was used as loading controls. Monoubiquitinated (6myc-HAS2 Ub_1_) and polyubiquitinated HAS2 (6myc-HAS2 poly-Ub) were quantified with ImageJ and normalized to total HAS2. The average of three experiments is presented±s.e.m. *P<0.05 and ***P<0.001, calculated with Student’s *t*-test.

To investigate whether the enzymatic activities of USP17 and USP4 are needed for their interaction with HAS2, we mutated the cysteine residue crucial for the catalytic activity of each of the DUBs. The catalytically inactive mutants C89S Flag-USP17 and C311S Flag-USP4 significantly de-ubiquitinated neither the wild-type 6myc-HAS2 nor the K190R mutant 6myc-HAS2, which is deprived of hyaluronan-synthesizing activity, but interacted with both wild-type and K190R mutant 6myc-HAS2. The ectopic expression of wild-type USP17 efficiently removed the polyubiquitinated chains from wild-type and K190R mutant 6myc-HAS2; a decrease in monoubiquitination was also observed (Figure 3a). Wild-type Flag-USP4 efficiently suppressed the monoubiquitination and also affected polyubiquitination of 6myc-HAS2 (Figure 3b).

### USP17 removes Lys63- and Lys48-linked polyubiquitin chains from HAS2, whereas USP4 preferentially removes monoubiquitin

**Figure 4:**
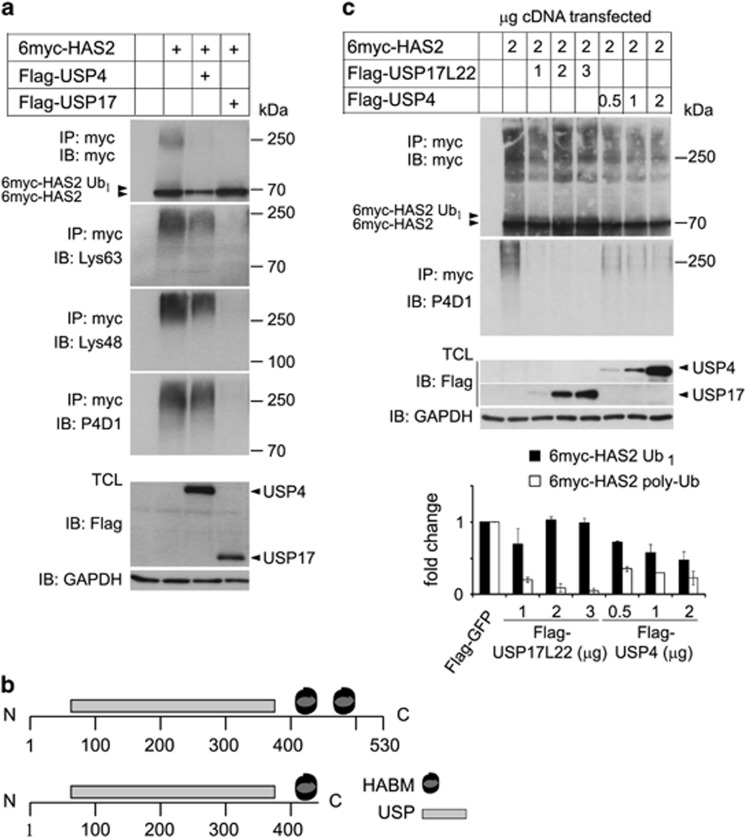
Lys48 and Lys63 polyubiquitin chains on HAS2 are efficiently removed by USP17, whereas USP4 also removes monoubiquitination. (**a**) HEK293T cells were co-transfected with 6myc-tagged HAS2 and Flag-tagged USP4 or USP17 cDNAs. 6myc-tagged empty vector and Flag-tagged vector encoding GFP were used as control and to equalize the DNA load. HAS2 was immunoprecipitated after denaturation and immunoblotting was performed with Lys63- or Lys48-specific polyubiquitin antibodies, as well as with P4D1 antibodies. Whole-cell lysates were probed with Flag-M2 antibody to verify DUB expression, and GAPDH was used as loading control. The data shown are a representative experiment out of three performed with similar results. (**b**) A schematic diagram of the USP17L22 and USP17 isoforms; the ubiquitin-specific protease domain (USP) and the two HABMs at positions 401–409 and 445–453, respectively, are depicted. (**c**) HEK293T cells were co-transfected with increasing amounts of Flag-USP17L22 (1–3 μg) or Flag-USP4 (0.5–2 μg), and 6myc-HAS2 (2 μg), and denaturated. Samples were subjected to immunoprecipitation with a myc antibody followed by immunoblotting with myc and P4D1 antibodies. A representative experiment out of two performed with similar results, and their quantification, is shown.

DUBs from the USP family are known to remove both Lys63- and Lys48-linked polyubiquitination chains.^52^ To investigate which type of ubiquitin chains are present on HAS2, and whether USP17 and USP4 are able to remove them, denaturated lysates from HEK293T cultures coexpressing 6myc-HAS2 and USP17 or USP4 were, after dilution, subjected to immunoprecipitation, followed by SDS–PAGE and immunoblotting with antibodies specific for Lys63- and Lys48-linked polyubiquitin chains. Both Lys63- and Lys48-linked polyubiquitination chains were detected on 6myc-HAS2. The co-transfection of Flag-USP17 efficiently removed both types of polyubiquitination, whereas Flag-USP4 removed partially both Lys63- and Lys48-linked polyubiquitin chains (Figure 4a).

Verification of the plasmid Flag-HA-USP17 by sequencing revealed a sequence encoding a truncated protein of 413 amino-acid residues that contained only the first hyaluronan-binding motif (HABM; Arg402-Lys410), but not the second (Lys445-Lys453). HABM motifs are found in hyaluronan-binding proteins, such as receptor for hyaluronan-mediated motility and CD44, and is defined as a R/K X_7_ R/K motif, where X is not an acidic residue and at least one of the seven amino-acid residues is basic.^63^ Therefore, we also investigated the de-ubiquitinase activity of a Flag-USP17L22 construct (Figure 4b), which encodes full-length protein and compared its specificity with USP4. USP4 and full-length USP17L22 were ectopically expressed at different concentrations together with 6myc-HAS2, and their hydrolytic specificities and capabilities were investigated under denaturating conditions followed by immunoprecipitation and immunoblotting. As shown in Figure 4c, USP17L22 already at the lowest expression level removed more than 80% of the polyubiquitination of HAS2, whereas USP4 was less efficient. On the other hand, expression of Flag-USP4 caused hydrolysis of ~50% of monoubiquitinated HAS2, whereas Flag-USP17L22 did not (Figure 4c). Our data suggest that USP17 preferentially removed polyubiquitination of HAS2, whereas USP4 preferentially removed its monoubiquitination and, partially, both Lys63- and Lys48-linked polyubiquitin chains (Figures 4a and c).

### USP17, but not USP4, stabilizes HAS2

**Figure 5:**
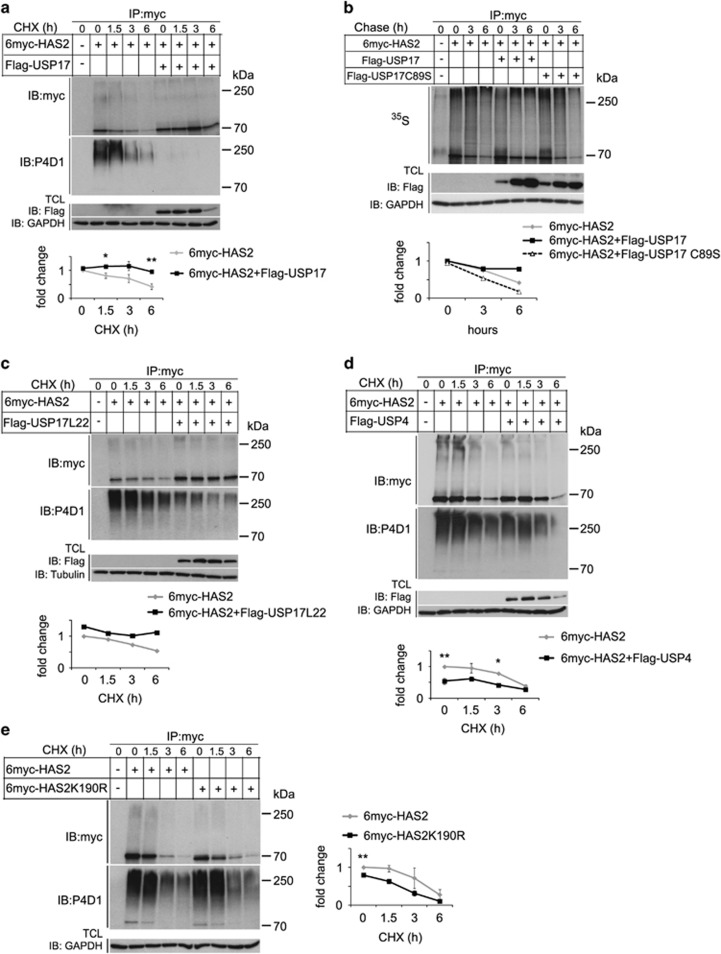
USP17, but not USP4, stabilizes 6myc-HAS2. HEK293T cells were transfected with 6myc-tagged HAS2 together with wild-type Flag-USP17 (**a**), wild-type or a catalytically deficient mutant of Flag-USP17 (**b**), Flag-USP17L22 (**c**), Flag-USP4 (**d**) or only HAS2 or the K190R mutant of HAS2 (**e**), and proteins were separated by SDS–PAGE. 6myc-tagged empty vector and Flag-tagged vector encoding GFP were used as control and to equalize the DNA load. Cells were left untreated or were treated with 20 μM cycloheximide (**a**–**e**) for different time periods and the half-life of 6myc-HAS2 was quantified. (**b**) Pulse-chase analysis of ^35^S-labeled 6myc-HAS2 was performed as described in Materials and methods, and the half-life of radioactively labeled 6myc-HAS2 was quantified. Quantifications in **b**, **c** depict a representative experiment out of three performed with similar results. Quantifications in (**a**, **d**, **e**) represent average±s.e.m. of three independent experiments. *P<0.05 and **P<0.01, calculated with Student’s *t*-test.

Because Lys48-linked polyubiquitination marks proteins for degradation in proteasomes, we explored further the importance of USP17 and USP4 for HAS2 stability, by an experiment in which *de novo* protein synthesis was blocked with cycloheximide. Denaturated lysates from HEK293T cells, co-transfected with 6myc-HAS2 or K190R mutant 6myc-HAS2, and USP4 or USP17, were prepared after different time periods and subjected to immunoprecipitation by a myc antibody, followed by immunoblotting with the myc antibody to detect HAS2 protein, or anti-P4D1 antibodies to detect polyubiquitination (Figure 5 and Supplementary Figure 2S). The intensities of the 6myc-HAS2 bands were quantified and the half-life of 6myc-HAS2 undergoing decay was calculated using Half Life Calculator, as described in Materials and methods. The analysis revealed that the half-life of 6myc-HAS2 was extended from 5 h to ~36 h upon coexpression with Flag-USP17 (Figure 5a, lower panel). Overexpression of the catalytically inactive mutant of Flag-USP17 (C89S) did not stabilize 6myc-HAS2 protein levels after cycloheximide treatment (Supplementary Figure 2S), consistent with the finding that it did not remove polyubiquitination from 6myc-HAS2 (Figure 3a and Supplementary Figure 2S).

To further consolidate these results, a pulse-chase assay was performed on cells incubated in a radioactive [^35^S]methionine/cysteine mix. After incubation in a medium supplemented with non-radioactive amino acids for the indicated time periods, 6myc-HAS2 was immunoprecipitated under denaturating conditions. Analysis by SDS–PAGE and autoradiography revealed that ^35^S-labeled 6myc-HAS2 levels were stabilized by coexpression of USP17, but not by coexpression of the catalytically inactive C89S mutant USP17 (Figure 5b). These results indicate that the USP17 subfamily proteins de-ubiquitinate HAS2 and thereby prevents its degradation in proteasomes. Similarly, overexpression of Flag-USP17L22 with 6myc-HAS2 stabilized the expression of HAS2 (Figure 5c).

Flag-USP4 decreased the monoubiquitinated form of 6myc-HAS2 (Figures 1a and 4a and c); we explored the possibility that this affects HAS2 expression and/or stability. Notably, coexpression of Flag-USP4 with 6myc-HAS2 caused a significant decrease in the level of HAS2; however, only a modest extension of the half-life of 6myc-HAS2 was observed, from 4.5 to 6 h, compared to control cells not transfected with USP4 (Figure 5d). Comparison of the protein levels and stability of wild-type and K190R mutant 6myc-HAS2 revealed that K190R mutant 6myc-HAS2 was expressed at lower steady-state levels, but had the same degradation pattern as wild-type 6myc-HAS2 (Figure 5e). These results suggest that USP4 and Lys190 of HAS2 are important for HAS2 protein expression, but not for stability, and that USP17 and USP4 differentially modulate HAS2 protein levels.

### Malignant cells express higher levels of HAS2, USP17 and USP4 compared to normal cells

**Figure 6:**
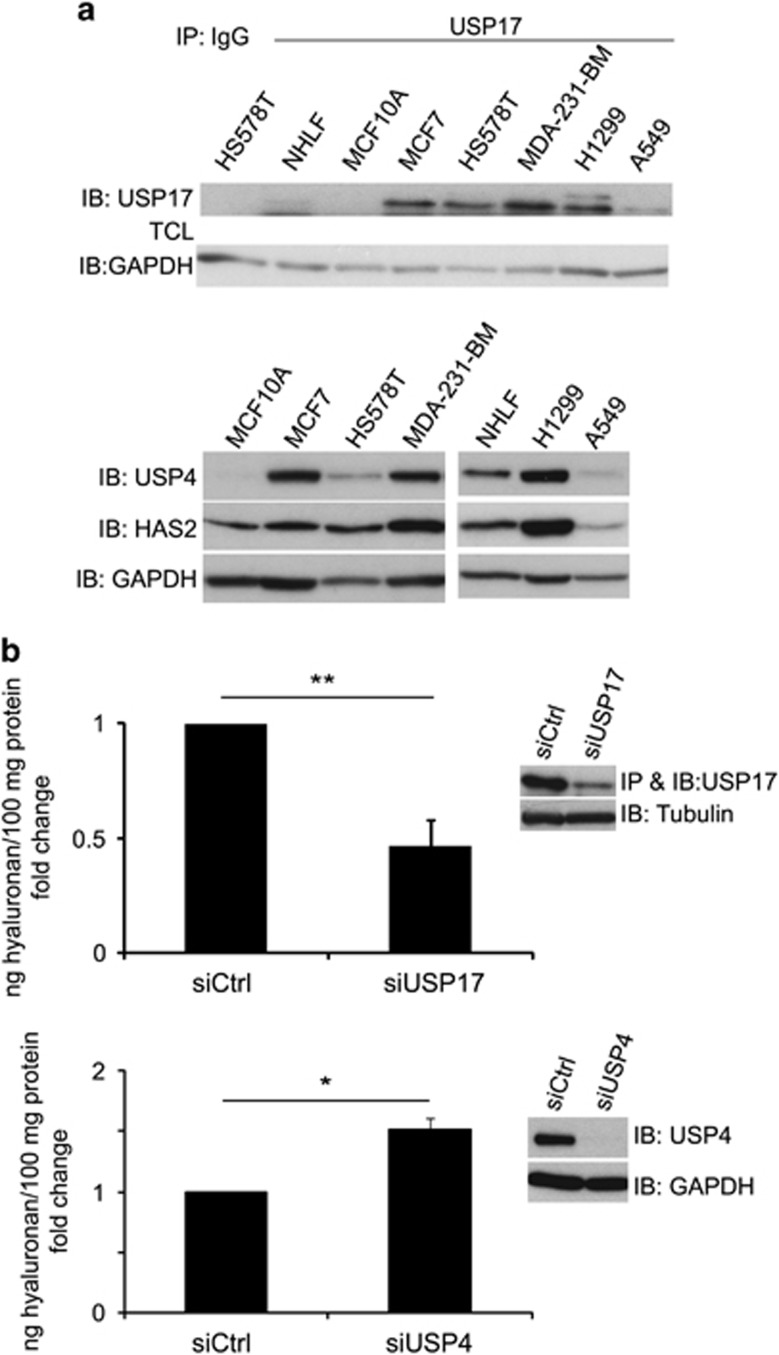
The levels of USP17, USP4 and HAS2 are higher in malignant compared to normal cells, and the knockdown of USP17 or USP4 differentially affects hyaluronan production. (**a**) Normal human lung fibroblasts (NHLF), breast epithelial cells (MCF10A), as well as breast cancer (MCF7, HS578T, MDA-MB-231-BM) and lung cancer (H1299 and A549) cells were subjected to immunoblotting by specific antibodies against USP17, USP4 and HAS2; the immunodetection of USP17 was preceeded by immunoprecipitation. Data represent one out of three experiments with similar results. (**b**) USP17 or USP4 were silenced in MDA-MB-231-BM, and the secreted hyaluronan in the culture media was measured by a hyaluronan assay. The inserts depict immunoblots with anti-USP17 or anti-USP4 antibodies to confirm their knockdown efficiencies. The graphs represent the average of three experiments±s.e.m. *P<0.05 and **P<0.01, calculated with unpaired Student’s *t*-test.

In order to investigate whether the expression levels of HAS2, USP17 and USP4 are correlated, we investigated their expressions in normal human lung fibroblasts and breast epithelial cells (MCF10A), as well as in breast cancer (MCF7, HS578T, MDA-MB-231-BM) and lung cancer (H1299 and A549) cells by immunoblotting. The immunodetection of USP17 was preceeded by immunoprecipitation, whereas that of USP4 and HAS2 was performed with immunoblotting of cell lysates by specific antibodies. As shown in Figure 6a, generally higher expressions of USP17, USP4 and HAS2 were detected in malignant breast and lung cells, compared to normal cells.

### Knockdown of USP17 decreases, whereas knockdown of USP4 increases hyaluronan production

We used the MDA-MB-231-BM cell line to investigate the importance of USP17 and USP4 for the HAS2-mediated hyaluronan production by silencing their expression and determining the levels of hyaluronan in conditioned media. The amounts of hyaluronan in the conditioned media were significantly decreased upon USP17 silencing, whereas depletion of USP4 resulted in a small but significant increase in hyaluronan production (Figure 6b).

### Deregulated expression of USP17, HAS2 and hyaluronan in neoplastic lung tissue

**Figure 7:**
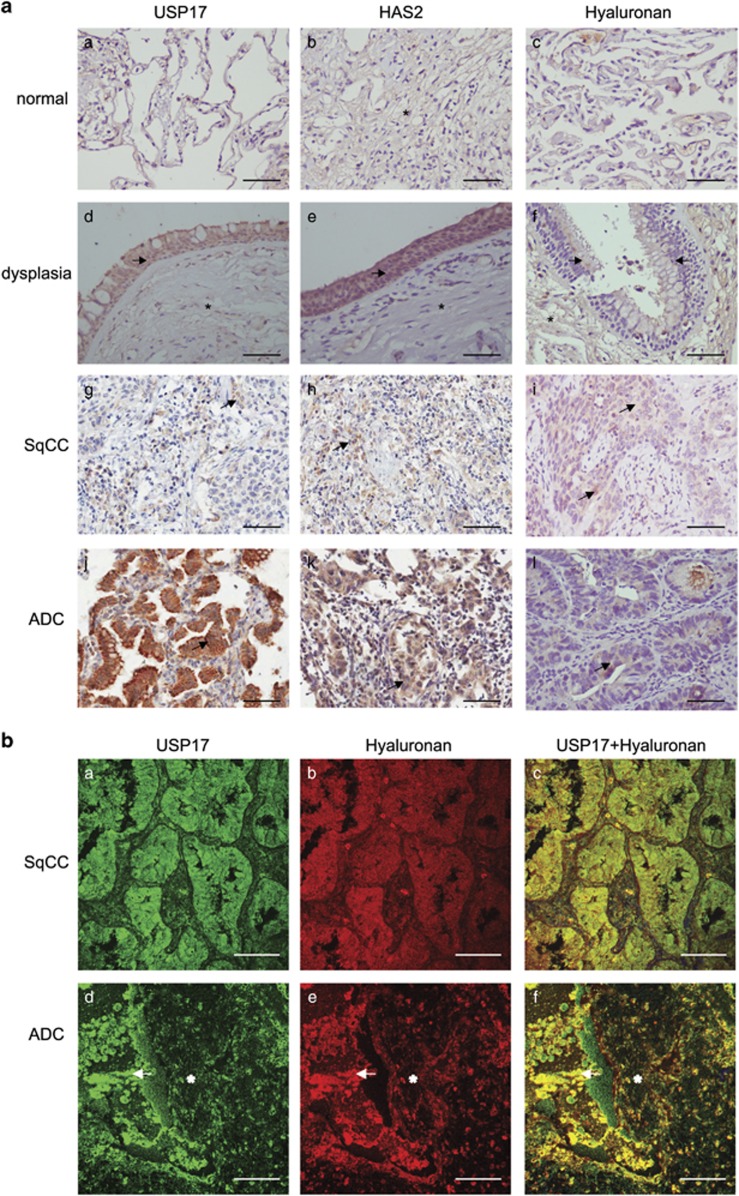
Detection of USP17, HAS2 and hyaluronan in non-small cell lung cancer (NSCLC) tissue. (**a**) Immunohistochemical stainings of USP17, HAS2 and hyaluronan in normal lung tissue (a–c), lung tissue showing dysplasia (d–f), SqCC (g–i) and acinar ADC (j–l). Arrows indicate cellular expression in epithelial cells of pre-neoplastic and neoplastic tissue, and asterisks indicate expression in stromal cells. Scale bar: 200 μm. (**b**) Immunofluorescence staining of USP17 and hyaluronan using specific anti-USP17 antibodies and a biotinylated globular domain of aggrecan, respectively, in SqCC (a–c) and acinar ADC (d–f). Arrows indicate cellular staining foci cell–stroma interface, asterisks indicate stromal tissue signal. Scale bar: 100 μm.

The expression of USP17, HAS2 and hyaluronan in pre-neoplastic and neoplastic lung tissue was investigated by immunohistochemistry (Figure 7a). The clinicopathological details of the lung patients have been described previously.^64^ The normal pulmonary epithelium had low expression of USP17, HAS2 and hyaluronan (29%, 16% and 25% of the cells stained, respectively; Table 1 and Figures 7Aa–c). USP17 and HAS2 expressions were more prominent in acinar adenocarcinoma (ADC) tumors (82% and 89%, respectively), and in dysplasia (68% and 75%, respectively), compared to squamous cell carcinoma (SqCC; 15% and 17%, respectively; Table 1 and Figures 7Ad, e, g, h j and k). Increased immunoexpression for hyaluronan was found in SqCC and ADC (42% and 58%, respectively), but not in dysplastic and normal lung (32% and 25%, respectively; Table 1 and Figures 7Ac, f, i and l). Thus, HAS2 and USP17 are significantly induced in lung carcinomas, in particular in ADC (*P*<0.005).

Because USP17 possesses two C-terminal HABMs, we investigated a possible co-localization of hyaluronan and USP17 in SqCC and acinar ADC (Figure 7b). Consistent with the immunohistochemical analyses, low amounts of hyaluronan and USP17 were observed in SqCC (Figures 7Ba and b), coincident with less co-localization as determined by confocal microscopy (Figures 7b and c). In contrast, a high expression of hyaluronan (48%) and high USP17 expression (78%) in acinar ADC were consistent with a higher degree of co-localization, as determined by confocal microscopy (Figures 7Bd–f).

## Discussion

By overexpressing individual DUBs, we identified several DUBs that reproducibly decreased monoubiquitination or polyubiquitination of HAS2, detected as mobility shifts in SDS–PAGE. Among the positive hits were USP17 and USP4. The de-ubiquitinase USP17 preferentially deconjugated polyubiquitin chains from HAS2, whereas USP4 significantly reduced the monoubiquitination of HAS2; thus, the two DUBs were found to selectively affect the activity and stability of HAS2.

The DUB3/USP17 subfamily of DUBs is conserved through species and has been implicated in regulation of cell fate and in diseases, such as autoimmunity and cancer.^65, 66^ USP17 regulates cell proliferation through modulation of Ras signaling, affecting the intracellular localization of Ras and other small GTPases.^67, 68, 69^ Furthermore, constitutive expression of USP17 blocks growth factor-dependent proliferation and initiates apoptosis,^70^ which may explain why our attempts to stably overexpress USP17 failed (data not shown). USP17 expression is tightly regulated during normal cell cycle progression; the expression is transiently increased at the G1–S phase transition, repressed at the S phase and became abundant again on entry into the G2–M phase and mitosis.^19^ In cells that are synchronized by double thymidine block and released, USP17 and HAS2 were found to be concomitantly induced at the G1–S and G2–M transitions (Figure 2d). The tightly regulated expressions of USP17 and HAS2 during cell cycle progression suggest an important role in the regulation of cell growth. Notably, localization studies revealed the presence of USP17 in the nucleus, in particular in the nucleoli, where also intracellular hyaluronan has been detected.^10, 15, 17^ Importantly, USP17 and other members of this subfamily with HABMs and RNA-binding motifs induce apoptosis and cell death of cancerous cells, whereas members without HABMs, such as USP17N, did not affect cell viability.^17, 18^ It has been demonstrated that HAS2-synthesized hyaluronan is abundantly induced during mitosis and the newly formed hyaluronan-enriched pericellular matrices facilitate the detachment of fibroblasts, keratinocytes and vascular smooth muscle cells during cell division.^71, 72, 73^

Hyaluronan is not only a prominent component of the pericellular and extracellular matrices, but is also found intracellularly, for example, in multiple myeloma and breast cancer cells, where it, most likely through its interaction with receptor for hyaluronan-mediated motility, associates with microtubules maintaining spindle pole integrity.^10, 11, 12, 15, 61^ The source of intracellular hyaluronan is not well understood. It is possible that a subpopulation is deposited in the cytoplasm, rather than first being secreted and then re-internalized.^74, 75^ Notably, the internalized extracellular and the endogenous intracellular hyaluronan exhibit different localization patterns.^10^

An interesting possibility, which remains to be elucidated, is that intracellular hyaluronan binds and cooperates with USP17 and/or other intracellular hyaluronan-binding proteins, affecting the structure of cytoskeleton or nuclear matrix during cell cycle progression. Interestingly, a strong co-localization between hyaluronan and USP17 was detected in acinar ADC in the cancer cell–stroma interface (Figure 7b). USP17 expression was negatively associated with glioma tumor grade, but increased in both SqCC and ADC patient tissues and correlated with recurrence and metastasis.^76, 77, 78^ A pro-tumorigenic mechanism of USP17 is to stabilize a key regulator of cellular division, phosphatase cdc25 (ref. 79) and a key EMT transcription factor, Snail1,^80, 81^ both of which are crucial for breast cancer progression and metastasis. Notably, excess hyaluronan production by HAS2 overexpression drives EMT by induction of Snail and Twist.^8^ Our data demonstrate that HAS2, hyaluronan and USP17 were expressed at high levels in pre-neoplastic lesions and acinar ADC (Figure 7a and Table 1), and at higher levels in metastatic breast cancer and lung cancer cell lines compared to normal cells (Figure 6a), suggesting that USP17-mediated stabilization of HAS2 resulting in increased hyaluronan synthesis, promotes non-small cell lung cancer and breast cancer tumor progression. As HAS2 has an established role in promoting EMT, partly through Snail,^8, 25^ USP17-mediated stabilization of both HAS2 and Snail might contribute to the pro-tumorigenic mechanisms of these proteins.

USP4 inhibits p53- and p53-mediated apoptosis, regulates NF-κB signaling pathway and modulates TGFβ signaling,^60, 82, 83, 84^ promoting breast cancer^85^ and lung adenocarcinoma invasiveness.^86^ We demonstrate here that both USP17 and USP4 are expressed at higher levels in breast and lung cancer cell lines compared to normal cells. Importantly, HAS2 was coexpressed in a similar pattern in the malignant cell lines, suggesting a correlation between HAS2, USP17 and USP4 protein levels. We observed that silencing of USP4 in breast cancer cells led to a small but significant increase in the amount of hyaluronan secreted into the culture media, which is consistent with an importance of Lys190 and possibly monoubiquitination for the regulation of hyaluronan synthesis by HAS2. The fact that the observed effects were not so dramatic could be because silencing of a single DUB may not be enough, as its action could be compensated for by other closely related DUBs, such as, for example, USP15.^87, 88^ USP4 was not as efficient in removing polyubiquitination chains from HAS2 as USP17, but has been shown to remove polyubiquitin chains from other targets, such as TGFβRI.^60^

Deregulated HAS2-synthesized hyaluronan is tightly connected to the malignant phenotype of solid tumors, such as breast cancer,^26, 27^ and the interaction between hyaluronan and its hyaluronan-binding proteins promotes cancer stemness;^8^ therefore, further elucidation of the mechanisms that regulate the ubiquitinylation of HAS2, and thus its stability and activity, is highly desired. Elucidation of the mechanism of how USP17 and HAS2 cooperate in the regulation of the cell cycle might be of therapeutic importance.

## Materials and methods

### Cell culture

HEK293T cells, MDA-MB-231-BM cells (a clone of the human breast cancer cell line MDA-MB-231 selected for its ability to metastasize to the bone^89^), HS578T, MCF7^90^ and the non-small cell lung cancer cell line A549 were routinely maintained in Dulbecco’s modified Eagle’s medium (DMEM, Sigma, Stockholm, Sweden) with 10% fetal bovine serum (Biowest, Lund, Sweden). The non-small cell lung cancer cell line H1299 was maintained in the RPMI-1640 medium (Sigma), supplemented with 10% fetal bovine serum and 2 mM L-glutamine. Normal human lung fibroblasts (2801-1; purchased from the Human Mutant Repository, Camden, NJ, USA) were cultured in DMEM (Sigma), supplemented with 10% fetal bovine serum and 5 μg/ml insulin (Sigma). MCF10 cells^91^ were routinely maintained in DMEM/F12 growth medium (Gibco, Life Technologies Europe BV, Stockholm, Sweden), supplemented with 5% horse serum (Invitrogen, Life Technologies Europe BV) 20 ng/ml epidermal growth factor (Peprotech, Preprotech Nordic, Stockholm, Sweden), 0.5 mg/ml hydrocortisone (Sigma), 100 ng/ml cholera toxin (Sigma) and 10 μg/ml insulin (Sigma).

### Constructs and vectors

A complementary (cDNA) library consisting of 69 Flag-HA-tagged DUBs in retroviral expression vectors (backbone vector MSCV-N-Flag-HA-IRES-PURO; Addgene, Cambrigde, MA, USA)^59^ was kindly provided by professor P ten Dijke, Leiden, the Netherlands. Cys89Ser and Cys311Ser mutants of Flag-HA-USP17 and Flag-HA-USP4, respectively, were generated by site-directed mutagenesis with the QuickChange Lightning Site-Directed mutagenesis kit (cat # 210518, Agilent Technology, Stockholm, Sweden) using the primers described in Supplementary Table 1S. Flag-USP17L22 in pcDNA3.1 (clone id B55533) was ordered from GenScript (Hong King, China, cat # Hu00062C). The 6myc-HAS2 and K190R mutant 6myc-HAS2 in pcDNA3 were described previously.^36^

### Screen of the DUB cDNA library

Each one of the Flag-HA-tagged DUB constructs of the cDNA library or the empty Flag-HA-GFP vector (0.5 μg) was co-transfected with 1 μg of the 6myc-HAS2 vector in HEK293T cells using polyethylenimine (Polysciences, Techtum Lab AB, Umeå, Sweden). After 24 h, cells were washed with ice-cold phosphate-buffered saline (PBS) and lysed in loading buffer containing 4% sodium dodecyl sulfate (SDS) and 20 mM dithiothreitol, sonicated and heated for 5 min at 95°C. Then, samples were resolved by SDS–PAGE.

### SDS–PAGE and immunoblotting

Cell lysates were analyzed by SDS–PAGE using 10% polyacrylamide gels, and proteins were transferred to a polyvinylidene fluoride (PVDF) membrane (Immobilon P, Merck Millipore, Solna, Sweden). Membranes were blocked by incubation in 5% milk in TBS-T (Tris-buffered saline containing 0.1% Tween 20) and incubated with primary antibodies, described in Supplementary Table 2S, diluted in TBS-T and supplemented with 1% bovine serum albumin and 0.02% NaN_3_. Proteins were visualized by chemiluminescence and exposed to X-ray film. Between each step, the membranes were washed 3 × 5 min in TBS-T. Band intensities were scanned and quantified by a densitometric software (ImageJ, 1.48d, NIH, Betheseda, MD, USA).

### Analysis of *in vivo* ubiquitination of HAS2

HEK293T cells were grown to in 10-cm cell culture dishes and then transfected with wild-type or mutant 6myc-HAS2 and wilt-type or mutant USP4 or USP17. After 24 h, cells were lysed in complete lysis buffer (1% SDS, 50 mM Tris, 150 mM NaCl, 2 mM EDTA, pH 8.0), supplemented with protease inhibitors (10 μg/ml leupeptin, 5 μg/ml aprotinin and 0.5 μg/ml Pefabloc) and phosphatase inhibitors (1 mM Na_3_VO_4_, 10 mM NaF). After sonication and heating for 5 min at 95 °C, samples were diluted 10-fold in dilution buffer (50 mM Tris, pH 8.0, 150 mM NaCl, 2 mM EDTA, 1% NP-40), supplemented with protease and phosphatase inhibitors. After centrifugation for 5 min at 10 000 r.p.m., 1 mg aliquots of protein of the supernatants were incubated with 20 μl of anti-c-Myc Agarose slurry (Thermo Scientific Pierce, Gothenburg, Sweden) for 1 h at 4 C with end-over-end mixing. The beads were then washed in dilution buffer, supplemented with 500 mM NaCl, followed by three washes in RIPA buffer (50 mM HEPES, pH 7.5, 150 nM NaCl, 10% glycerol, 0.1% SDS, 1% NP-40, 0.5% Na-deoxycolate), supplemented with protease and phosphatase inhibitors. The adsorbed proteins were eluted by heating at 95 °C for 5 min in SDS sample buffer, followed by SDS–PAGE and immunoblotting with P4D1 antibodies to detect ubiquitinated HAS2, and c-Myc antibodies to detect HAS2 expression.

### Crude membrane extraction and co-immunoprecipitation

Confluent monolayers of HEK293T cells in 10-cm culture dishes were washed in ice-cold PBS and scraped into sucrose buffer (10 mM HEPES, pH 7.1, 0.25 M sucrose, 1 mM dithiothreitol), supplemented with protease and phosphatase inhibitors. Samples were then sonicated 6 times 1 min, and then subjected to ultracentrifugation in a Beckman Ultra-Centrifuge L8-M with a SW50.1 rotor at 40 000 r.p.m., for 1 h at 4 °C. The pelleted crude membrane fractions were resuspended in TBS/Ca^2+^ buffer (25 mM Tris, 150 mM NaCl, 1 mM CaCl_2,_ pH 7.4), supplemented with 0.1% SDS, 0.5% NP-40, and protease and phosphatase inhibitors, for a minimum of 1 h at 4 °C. Insoluble material was removed by centrifugation at 10 000 r.p.m. for 10 min at 4 °C, where after the protein concentration in the supernatant was measured with BCA protein assay reagent kit (Pierce). Aliquots of 500 μg of protein were incubated with 3 μg of anti-Myc antibodies, 30 μl of anti-Flag-M2 magnetic beads slurry (Sigma) or mouse IgG control antibody overnight at 4 °C. The c-myc antibody immunoprecipitated complexes and control IgG were captured by 30 μl protein G-Sepharose beads (Amersham Biosciences) by incubation with end-over-end mixing for 1 h at 4 °C. Beads were washed four times in TBS/Ca^2+^ buffer, and proteins were then eluted by heating for 5 min at 95 °C in SDS sample buffer. Samples were resolved by SDS–PAGE and analyzed by immunoblotting.

### Proximity ligation assay

MDA-MB-231-BM cells in eight-chamber culture slides (Falcon) were washed in ice-cold PBS, fixed for 10 min in ice-cold acetone and washed again in PBS; *in situ* PLA was then performed at the PLA proteomics facility, SciLife Lab, Uppsala, Sweden, using the antibodies described in Supplementary Table S2. The endogenous complexes, visualized by fluorescent dots, between HAS2 and USP17, or HAS2 and USP4, were counted by Duolink ImageTool (Olink Bioscience). As negative controls, one of the primary antibodies was omitted or rabbit IgG isotype control was used instead of anti-USP17.

### Propidium iodide staining and analysis by FACS

MDA-MB-231-BM cells were blocked in G1/S by double thymidine (2 mM) block, trypsinized and fixed in 70% ethanol for 30 min at 4 °C. Then, cells were incubated with Ribonuclease A (100 μg/ml) and propidium iodide (100 μg/ml), both from Sigma, for 45 min on ice. Samples were analyzed by flow cytometry using a BD LSR Fortessa and the BD FACSDiVa version 8.0 software. The cell cycle progression data were analyzed by ModFit LT (Becton Dickinson AB, Stockholm, Sweden).

### Analysis of 6myc-HAS2 stability

The turnover of 6myc-HAS2 or K190R mutant 6myc-HAS2, in the absence or presence of USP17/USP17L22, the catalytically inactive C89S mutant USP17 or USP4, was determined by immunoblotting. Protein synthesis was inhibited by treatment with 20 μM cycloheximide (Sigma), followed by incubation for the indicated time periods. The relative 6myc-HAS2 band intensities at 0 h (*N*_0_) and later time points (*N*_t_) were quantified, and a half-life was calculated using the formula *N*_t_=*N*_0_(1/2)^*t/t*1/2^ (www.calculator.net/half-life-calculator).

The stability of HAS2 was also studied by a pulse-chase assay using metabolic labeling with ^35^S-methionine/cysteine. HEK293T cells in 6-cm dishes were transfected with the indicated plasmids using Lipofectamine 3000 (Invitrogen, Carlsbad, CA, USA). After 24 h, cells were washed in PBS and starved for 30 min in methionine/cysteine (Met/Cys)-free culture medium (DMEM, Gibco), supplemented with 10 mM HEPES (Sigma). Cells were then incubated for 30 min with 150 μCi [^35^S]Met/Cys mix (Easy-tag protein labeling mix; Perkin Elmer, Waltham, MA, USA) and chased in DMEM medium supplemented with 10% fetal bovine serum, 2 mM methionine and 2 mM cysteine, for the indicated time periods. Cells were harvested in PBS, snap-frozen in liquid nitrogen and kept at −80 °C until lysis; the amount of HAS2 was determined by immunoprecipitation, followed SDS–PAGE and autoradiography.

### Hyaluronan assay

The amount of hyaluronan in conditioned media of cultured cells was quantified by an assay based on the specific interaction of hyaluronan with the G1 global domain of aggrecan, immobilized to 96-well microtiter plate (MaxiSorp Nunc-Immuno plates, Thermo Fischer Scientific, Gothenburg, Sweden).^36^ The amount of hyaluronan in the samples was normalized to the amount of cellular protein.

### Transient silencing of USP4 and USP17 and stable knockdown of HAS2 in MDA-MB-231-BM cells

Short interfering RNAs (siRNAs; 20 nM) against human USP4 (trilencer-27 cat #SR305038 sequence A or C, Origene, Rockville, MD, USA), control siRNA (# SR30004, Origene) or ON-TARGET plus Human USP17L2 siRNAs (cat. #J-027332-11, #J-190062-05, Dharmacon, Thermo Fischer Scientific) were transiently transfected into MDA-MB-231-BM cell cultures by Silentfect (Bio-Rad) according to the manufacturer’s instructions; USP17 siRNAs (30 nM each time) were transfected two times. Fresh media was added after 72 or 96 h to USP4- or USP17L2-depleted cell cultures, respectivey, followed by collection of the MDA-MB-231-BM cell conditioned media 24 h later. Cells were lysed in RIPA buffer supplemented with protease and phosphatase inhibitors. Equal amounts of protein were subjected to SDS–PAGE, followed by immunoblotting.

Stable knockdown of HAS2 in MDA-MB-231-BM cells was performed by infecting cells with lentiviral transduction particles with shRNA targeting human HAS2 (MISSION, cat. # SHCLNV), or control shRNA (cat.# SHC002V) with a multiplicity of infection of 5. Transduced cells were selected by culturing in a growth medium supplemented with 1 μg/ml puromycin (Calbiochem, Merck, Germany). The constructs had the following sequences:

#3,CCGGGAATATCTCAGATGGCTAAACCTCGAGGTTTAGCCATCTGAGATATTCTTTTTTG;

#4,CCGGTACGATTCCTGGATCTCATTCCTCGAGGAATGAGATCCAGGAATCGTATTTTTTG.

### Immunohistochemistry and immunofluorescence

The lung specimens used in this study were obtained from the tumor bank of the São Paulo University Hospital, and the study was approved by the Institutional Ethics and Scientific Committee of São University Hospital (0171/09-hospital das Clinicas, Sao Paulo, Brazil).^64^

Immunohistochemistry for USP17, HAS2 and hyaluronan in pre-neoplastic and neoplastic lesions (Table 1) was performed essentially as described.^64^ The slides were incubated overnight with antibodies described in Supplementary Table S2, and staining for USP17 was visualized by the Novolink Max Polymer (RE-7260-K) and for HAS2 by ImmPRESS Kit (Vector Laboratories, Burlingame, CA, USA). Hyaluronan was visualized essentially as described.^92^ Chromogen solution (DAB; Sigma Diagnostics) was applied and the sections were counterstained with hematoxylin before mounting in a xylene-based medium. Samples were quantified using an Automated Cellular Imaging Systems (ACIS; DAKO-Agilent Technologies, Carpinteria, CA, USA), as described.^64^

Immunofluorescence staining for hyaluronan and USP17 was performed by using the biotinylated globular domain of aggrecan (1:150; kindly donated by Dr Helena Nader from the Biochemistry Department, Escola Paulista de Medicina, Sao Paulo, SP, Brazil) and anti-USP17 antibody (1:50; ab188236), respectively. Colocation was evaluated using confocal microscopy Zeiss LSM-410 (Carl Zeiss AG, Oberkochen, Germany). For negative controls, sections were treated with hyaluronidase or incubated with PBS instead of the primary antibody, respectively. The sections were then incubated with the secondary antibodies, that is, streptavidin ALEXA 546 and goat anti-rabbit ALEXA 488 (1:400; Invitrogen) for 3 h. Nuclei were stained with 496-diamidino-2-phenylindole, dihydrochloride for 30 min (Invitrogen; 1:300). Serial optical sections were performed with the Simple 32 C-imaging computer software (LSM Image Browser software, Carl Zeiss), collected at 0.6 mm with a 660 Plan. Apo lens and a scan zoom of 62. Images were processed and reconstructed using the US National Institutes of Health Image software (Bethesda, MD, USA).

### Statistical analysis

Statistical analysis was performed by analysis of variance for multiple comparisons, followed by appropriate *post hoc* tests, such as the Bonferroni test and by the Student's *t*-test for comparison of two variables between groups. The statistical program used was SPSS 18.0 (SPSS Inc., USA). A *P*-value<0.05 was considered to be significant.

For non-patient material, samples were assumed to be unpaired and a two-tailed Student’s *t*-test was used to calculate significance. A *P*-value<0.05 was considered significant.

## Figures and Tables

**Table 1:** Staining intensity of USP17, HAS2 and hyaluronan in pre-neoplastic and neoplastic lung tissue

*Antigens*	*Pre-neoplastic tissue*	*Neoplastic lesion*	*Intensity (means±s.d.*^a^)	P*-value compared to normal*
*USP17*				
	Normal		29.9±16.3	
	Dysplasia		68.3±22.3	0.005
		SqCC	15.8±15.0	0.06
		ADC	82.6±12.0	0.001
				
*HAS2*				
	Normal		16.5±8.5	
	Dysplasia		75.1±9.4	0.01
		SqCC	17.7±17.4	>0.5
		ADC	89.5±18.2	0.001
				
*Hyaluronan*				
	Normal		25.2±10.5	
	Dysplasia		32.4±13.5	0.09
		SqCC	42.9±12.4	0.05
		ADC	58.0±14.4	0.02

Abbreviations: ACIS, Automated Cellular Imaging System; ADC, acinar adenocarcinoma; SqCC, squamous cell carcinoma.

SqCC, *n*=25; ADC, *n*=31; normal, *n*=12; dysplasia, *n*=10.

a Calculated with the software ACIS III as described.^63^ A *P*-value<0.05 was considered to be significant.
