# Supplementary material for: The deubiquitinating enzymes USP4 and USP17 target hyaluronan synthase 2 and differentially affect its function
Source: Oncogenesis. 2017 Jun 12;6(6):e348–. doi: 10.1038/oncsis.2017.45 (PMC5519194; doi:10.1038/oncsis.2017.45)
Supplement: Supplementary Material [file oncsis201745x1.docx]

**Supplementary Material**

**Supplementary Table 1S. Mutagenic primers for generation of C89S mutant USP17 and C311S mutant USP4**

| **Gene primer sequences** |
| --- |
| C89S mutant USP17 Forward: 5'-ggaagcgttcacgtagctggtatttcccatattct-3  Reverse: 5’agaatatgggaaataccagctacgtgaacgcttcc-3' |
| C311S mutant USP4 Forward: 5'-gcggagttcatgaagctggtgtttcccaggttt-3'  Reverse: 5'-aaacctgggaaacaccagcttcatgaactccgc-3' |

**Supplementary Table 2S. Antibodies used in this study for immunoprecipitation (IP), immunoblotting (IB), proximity ligation assay (PLA), and immunohistochemical (IHC) and immunofluorescence (IF) staining**

| **target** | **use** | **supplier** | **designation** | **dilution or concentration** |
| --- | --- | --- | --- | --- |
| c-Myc peptide | IB, IP | Santa Cruz | sc-40 9E10 | 1:500 |
| ubiquitin | IB | Santa Cruz | sc-8017 | 1:500 |
| USP17L | IB | Santa Cruz | sc-103318 | 1:500 |
| DUB3/USP17 | PLA | Abcam | ab188236 | 1 μg/ml |
| cyclin D1 | IB | Santa Cruz | sc-8396 | 1:200 |
| HAS2 | IB | Santa Cruz | sc-514737 | 1:500 |
| HAS2 | PLA, IHC | Santa Cruz | Sc-34067 | 1 μg/ml |
| ubiquitin Lys-48 | IB | Millipore | 05-1307 | 1:1000 |
| ubiquitin Lys-63 | IB | Millipore | 05-1308 | 1:1000 |
| GAPDH | IB | Cell Signaling | D16H11 | 1:20 000 |
| cyclin B1 | IB | Cell Signaling | 4138S | 1:1000 |
| USP4 | IB | Bethyl Laboratories | A300-830A | 1 μg/ml |
| USP4 | PLA | Sigma-Aldrich | HPA018499 | 1 μg/ml |
| Flag-M2 | IB | Sigma-Aldrich | F-3165 | 5 μg/ml |
| HRP-conjugated mouse IgG | IB | Invitrogen |  | 1:10 000 |
| HRP-conjugated rabbit IgG | IB | Invitrogen |  | 1:10 000 |
| mouse IgG control | IP | Santa Cruz | sc-2025 | 1:500 |
| rabbit IgG control | PLA | Abcam | ab27478 | 1 μg/ml |





**Supplementary Figure 1S: DUB cDNA expression screen to identify HAS2 deubiquitinases.**

HEK293T cells were co-transfected with 6myc-tagged HAS2 (2 μg) and individual Flag- HA- tagged DUB cDNAs (1 μg). 6myc-tagged empty vector and Flag-tagged vector encoding GFP

were used as control and to equalize the DNA load. Denaturated samples were subjected to SDS-PAGE followed by immunoblotting with P4D1 antibodies to detect poly-ubiquitination and myc antibodies to detect 6myc-HAS2. Antibodies against Flag were used to detect the expression of the individual DUBs. * indicates the expressed DUB.





**Supplementary Figure 2S: USP17, but not the catalytically inactive USP17 C89S, stabilizes 6myc-HAS2.**

HEK293T cells were co-transfected with 6myc-tagged HAS2 and Flag-USP17 or a catalytically deficient mutant of Flag-USP17 (C89S). Cells were left untreated or treated with 20 μM cycloheximide for up to 6h, and the stability of 6myc-HAS2 was quantified. Cell lysates were then prepared and subjected to immunoprecipitation with a myc antibody, followed by SDS-PAGE and immunoblotting with antibodies against myc and poly-ubiquitin (P4D1). The 70 kDa 6myc-HAS2 band was quantified (lower panel). 6myc-tagged empty vector and Flag-tagged vector encoding GFP were used as control and to equalize the DNA load.
